# Identifiability and Identification of Trace Continuous Pollutant Source

**DOI:** 10.1155/2014/215104

**Published:** 2014-04-27

**Authors:** Hongquan Qu, Shouwen Liu, Liping Pang, Tao Hu

**Affiliations:** ^1^College of Information Engineering, North China University of Technology, Beijing 100144, China; ^2^China School of Aviation Science and Engineering, Beijing University of Aeronautics and Astronautics, Beijing 100191, China; ^3^China Aero-Polytechnology Establishment, Beijing 100028, China

## Abstract

Accidental pollution events often threaten people's health and lives, and a pollutant source is very necessary so that prompt remedial actions can be taken. In this paper, a trace continuous pollutant source identification method is developed to identify a sudden continuous emission pollutant source in an enclosed space. The location probability model is set up firstly, and then the identification method is realized by searching a global optimal objective value of the location probability. In order to discuss the identifiability performance of the presented method, a conception of a synergy degree of velocity fields is presented in order to quantitatively analyze the impact of velocity field on the identification performance. Based on this conception, some simulation cases were conducted. The application conditions of this method are obtained according to the simulation studies. In order to verify the presented method, we designed an experiment and identified an unknown source appearing in the experimental space. The result showed that the method can identify a sudden trace continuous source when the studied situation satisfies the application conditions.

## 1. Introduction

A pollution incident has the potential to create significant harm on numbers of people, especially when it happens in an enclosed ventilation space such as a cabin in a manned spacecraft, submarine, or aircraft. It is urgent to develop related technology to efficiently locate a sudden pollutant source so that control actions can be taken rapidly.

Pollutant source identification is a process that searches a source term reversely by using limited information. It usually consists of detecting the initial emission time, estimating emission strength and finding the source location. However, inverse identification for a source is an extremely difficult challenge in the research field of inverse problems, namely, for the complexity in the coupling of source position, strength and emission time, and the incompleteness and uncertainty of observed data. With the improvement of computer processing capacity, the identification of an accidental pollutant source is becoming a more popular topic in some fields, such as research of pollution in atmospheric environment, water environment, enclosed spaces, and porous media. Many researchers attempt to use some potential method to detect contaminant source [1, 2].

Most of the methods used retrospective methods. These methods use the sensor observed data to estimate the unknown pollutant source. These methods can be further classified into analytical, optimization, probabilistic, and backward computational fluid dynamics (CFD) methods.

The analytical method can identify a pollutant source based on an analytical solution of a velocity field and a measured concentration distribution. It is often applied in an atmospheric environment or on surface water where a steady flow field is present [3–5].

The optimization method can search for the optimal value by comparing the measured pollutant concentration data with the corresponding calculated data. The linear or nonlinear optimization and the maximum likelihood method are widely adopted. This method requires a large amount of direct calculation and this may lead to a heavy computation load. This method is successfully used to identify the source in an atmospheric environment and in underground water [6–10]. Although this method is quick and efficient for simple situations, it is difficult to obtain the analytical solution for a more complicated flow field. In order to improve the robustness of this method, some researchers attempted to use sensitivity analysis in order to reduce the uncertainty [11, 12].

The probabilistic method uses probability theory to calculate the possibility that a pollution source appears at a certain position [13, 14]. By using this method, the ill-posed problem of source identification may be transformed into a well-posed problem in the extended statistical space [15, 16]. Many researchers have tried to apply this method to identify an indoor pollutant source and to optimize the placement of sensors [17–23]. Though there are some shortcomings of this method, such as the complexity of the solution of the adjoint state equations, and the fact that the uncertainty of the model cannot be overcome completely, it is indeed a promising method for identifying a source.

The backward CFD method can inversely evaluate the source information by using a negative time step and a reverse flow field. The inverse CFD model is ill-posed, so regularization technique and stabilization technique should be used to improve its solution stability [24]. Some researchers carried out several studies to improve this stability [25–28]. Currently, the combination of the probabilistic method and the backward CFD method is a significant advance in the identification of a pollutant source in an enclosed space [18–20]. They used backward PDF combined with the quasireversibility method and pseudoreversibility method, respectively, to identify an instantaneous source by using the accurate observed data of a single sensor. Although this research requires an accurate velocity field and exact sensor measured data, it has proved to be promising.

The above endeavors have been promoting the development of source identification. Actual pollutant source emission sometimes is a continuous and not instantaneous process, and the measured data is influenced by sensor noise. So, we develop a method to identify a sudden continuous emission source in a steady velocity field that remains unchanged or little changed during a source emission process by using single sensor information with noise. The application conditions for this method are presented and its assumptions are discussed in detail.

## 2. Model of Pollutant Transport Process

Assume that a source emits pollutant in a closed space with a constant temperature condition and a small viscous stress and then a gaseous pollutant transport process by air is governed by the ADE and momentum equations [29]:

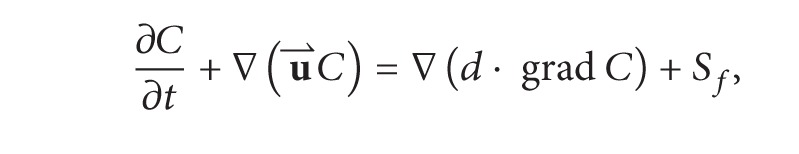
(1)

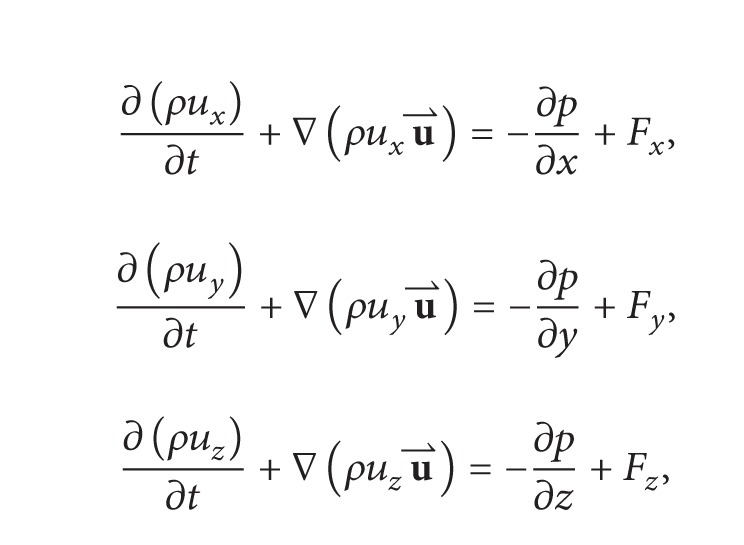
(2)
where *C* is the pollutant mass concentration, mg/m^3^; *t* is the forward time, s; $\overset{⃑}{u}$ is the velocity vector, m/s; *u*_*x*_, *u*_*y*_, and *u*_*z*_ are the velocity components in *x*, *y*, and *z* directions of $\overset{⃑}{u}$; *d* is the diffusion coefficient, m^2^/s; *S*_*f*_ is the pollutant source which is a function of (*p*, *S*, *t*_*e*_); *p* is the source position; *S* is the emission strength, mg/(m^3^ *·*s); *ρ* is the fluid density, kg/m^3^; *p* is the fluid pressure on the micro element; and *F*_*x*_, *F*_*y*_ and *F*_*z*_ are the body forces in *x*, *y*, and *z* directions, N.

We can obtain an accurate solution of the concentration field by solving the ideal gas equation and (1)~(2). However, this simultaneous solution is very complex and time-consuming. It will become even more difficult to identify a pollutant source reversely because it should solve $\overset{⃑}{u}$ and *S* together by using the partially known *C*.

In order to avoid this complex and time-consuming calculation process, we assume the studied situation satisfies the following two conditions before and after a source appears: (1) $\overset{⃑}{u}$ is large enough; (2) *ρ* is approximately uniform; and (3) they remain unchanged or little changed.

With these two assumptions, a pollutant source identification process can be transformed into a relatively simple inverse problem because (2) can be neglected and only (1) needs to be considered. We can estimate the three parameters (*p*, *S*, and *t*_*e*_) of the source by solving (1) reversely when $\overset{⃑}{u}$ and a part of *C* are known.

Equation (1) can be solved numerically. When the pollutant concentration distribution at time *t* is known, the concentration distribution sequent at time *t* + 1 can be obtained as follows:

(3) $$\begin{array}{c} AC_{}^{t+1}=aC_{}^{t}+S_{f}, \end{array}$$

where *C* ^*t*+1^ and *C* ^*t*^ are the column vectors of the pollutant concentration for each grid node at time *t* + 1 and *t*, respectively; **a** is the coefficient matrix of *C* ^*t*^; and **A** is a diagonally dominant banded sparse matrix; hence, it must have the inverse matrix **A** ^−1^:

(4) $$\begin{array}{c} C_{}^{t+1}=A^{-1}aC_{}^{t}+A^{-1}S_{f}\text{.} \end{array}$$

**A** can be uniquely determined when the computational zone, the mesh, and the discretization scheme are known. We chose the power-law scheme to solve the discretized equations of pollutant ADEs in this paper.

## 3. Source Identification Method

Based on the above assumptions about $\overset{⃑}{u}$ and *ρ*, we will develop a method to identify a sudden trace pollutant source by using a single sensor with noise.

A sensor can detect a sudden pollution when its observed concentration rises upon a given threshold value, *C*th. We define two time, *t*_*e*_ and *t*_*p*_. *t*_*e*_ is the initial emission time when a source begins to emit a pollutant. *t*_*p*_ is the detecting time when a sensor takes to detect the existence of the pollutant. There is a delay time *t*_*d*_ and *t*_*d*_ = *t*_*p*_ − *t*_*e*_.

**Figure 1:**
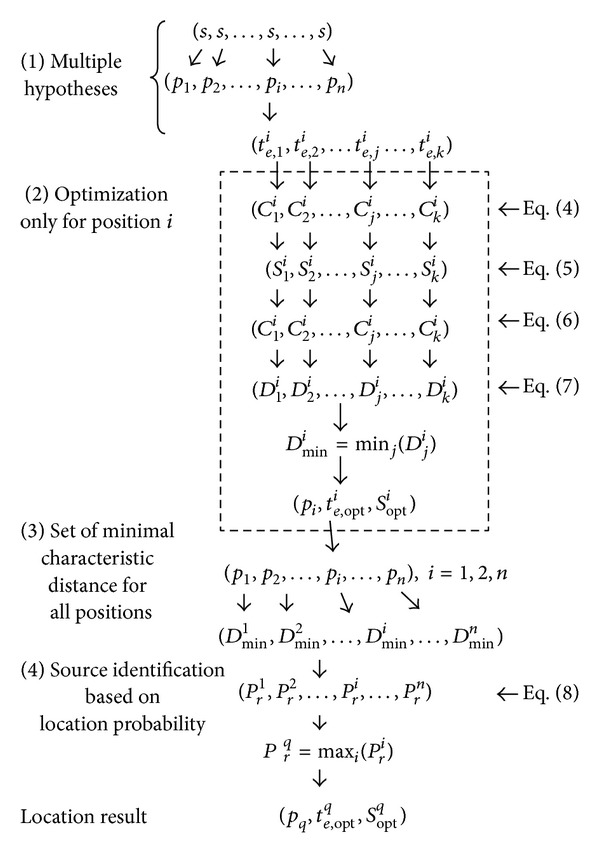
Implementation steps for a source identification.

An identification process can be conducted after *t*_*p*_and *C*_sensor_ are known. We use the idea of multiple parameter multiple hypothesis optimization to identify the unknown three source parameters (*p*, *S*, and *t*_*e*_) of source. The measured concentration sequence of sensor in the time interval [*t*_*p*_, *t*_check_] is specially taken out from *C*_sensor_ and defined by *C*_senCheck_ for the identification. There are four required steps to implement this process, as shown in Figure 1.

*(A) Multiple Hypothesis of Source Position.* A set of hypotheses of source position, (*p*_1_, *p*_2_,…, *p*_*i*_,…*p*_*n*_) is established firstly. It includes all possible source positions. The second hypothesis assumes that a virtual pollutant source with a constant emission strength, *s*, will appear on every possible hypothetical position *p*_*i*_, respectively. For a certain *p*_*i*_, a set of hypotheses, (*t*_*e*,1_^*i*^, *t*_*e*,2_^*i*^,…, *t*_*e*,*j*_^*i*^,…, *t*_*e*,*k*_^*i*^), will be established thirdly. If the backward searching time index is *j*, then *t*_*es*,*j*_^*i*^ = *t*_*p*_ − *j* × Δ*t*.

*(B) Optimization Only for p*_*i*_. The optimal three parameters of source only for *p*_*i*_ can be searched with the objective function of minimum characteristic distance. It includes the following steps.

(a) When $\overset{⃑}{u}$ and *d* are known, the hypothetical measured concentration sequence, (*c*_1_^*i*^, *c*_2_^*i*^,…, *c*_*j*_^*i*^,…, *c*_*k*_^*i*^), at sensor position for *p*_*i*_ can be calculated by forwardly solving (4) in [*t*_*p*_, *t*_check_] with the assumption of (*p*_*i*_, *s*, *t*_*e*,*j*_^*i*^).
(b) Assume that $\overset{⃑}{u}$ remains unchanged or little changed during the pollutant emission process and then the steady-state observed concentration is linear with respect to emission rate [23]; hence, the approximate relative emission strength *S*_*j*_^*i*^ can be obtained as follows:
  (5) $$\begin{array}{c} S_{j}^{i}=\frac{S}{s}=\frac{\int_{t_{p}}^{t_{\text{check}}}\left(C_{\text{senCheck}}-C_{0}\right)dt}{\int_{t_{p}}^{t_{\text{check}}}\left(c_{j}^{i}-C_{0}\right)dt}, \end{array}$$
  where *s* is the constant emission strength of the virtual pollutant source, mg/(m^3^·s); *S* is the estimated emission strength, *S* = *S*_*j*_^*i*^ · *s*, mg/(m^3^·s); *S*_*j*_^*i*^ is the estimated relative emission strength with the assumption of (*p*_*i*_, *s*, *t*_*es*,*j*_^*i*^); and *C*_0_ is the initial concentration, mg/m^3^.
(c) After (*S*_1_^*i*^, *S*_2_^*i*^,…, *S*_*j*_^*i*^,…, *S*_*k*_^*i*^) are calculated by using (5), the concentration at sensor position in the time interval [*t*_*p*_, *t*_check_] can be calculated forwardly by using (4) and is denoted as the hypothetical measured concentration sequence of sensor *C*_*j*_^*i*^:
  (6) $$\begin{array}{c} C_{j}^{i}=S_{j}^{i}\left(c_{j}^{i}-C_{0}\right)+C_{0} \end{array}$$
  and (*C*_1_^*i*^, *C*_2_^*i*^,…, *C*_*j*_^*i*^,…, *C*_*k*_^*i*^) can be obtained with (6).
(d) We defined a characteristic distance to describe the similarity degree between *C*_*j*_^*i*^ and *C*_senCheck_ in [*t*_*p*_, *t*_check_]:
  (7) $$\begin{array}{c} D_{j}^{i}=\sqrt{\overset{t_{\text{check}}}{\underset{t=t_{p}}{\sum }}{\left(C_{j,t}^{i}-C_{\text{senCheck},t}\right)}^{2},} \end{array}$$
  where *D*_*j*_^*i*^ is the characteristic distance between *C*_*j*_^*i*^ and *C*_senCheck_.
(e) After the set of (*D*_1_^*i*^, *D*_2_^*i*^,…, *D*_*j*_^*i*^,…, *D*_*k*_^*i*^) has been calculated with (7), we define the minimum characteristic distance *D*_min⁡_^*i*^ = min⁡_*j*=1,2,…,*k*_(*D*_*j*_^*i*^). *D*_min⁡_^*i*^ will be an objective function to search the optimal three parameters of source only for the position *p*_*i*_. Let “opt” express the optimal one in the set of (*j* = 1,2,…, *k*) and then *t*_*e*,opt_^*i*^ = *t*_*p*_ − *j*_opt_^*i*^ × Δ*t* and *S*_opt_^*i*^ can be optimized accordingly to *D*_min⁡_^*i*^. Now, the optimal result for the hypothetical position *p*_*i*_ is (*p*_*i*_, *t*_*e*,opt_^*i*^, *S*_opt_^*i*^).

*(C) Minimum Characteristic Distance Set.* Similarly, the set of minimum characteristic distance, (*D*_min⁡_^1^, *D*_min⁡_^2^,…, *D*_min⁡_^*i*^,…, *D*_min⁡_^*n*^), can be finally obtained for the other hypothetical positions in the set of (*p*_1_, *p*_2_,…, *p*_*n*_).

*(D) Source Identification Based on Location Probability. *A location probability is established to clearly point out the most probable source positions after {*D*_min⁡_^1^, *D*_min⁡_^2^, *D*_min⁡_^3^,…, *D*_min⁡_^*n*^} has been obtained. We use the location probability to show the probability of a source appearing at the *i*th assumed position [14]:

(8) $$\begin{array}{c} P_{r}^{i}=e^{-{\left(D_{\min}^{i}/n\sigma \right)}^{2}}\times {\left(\overset{n}{\underset{i=1}{\sum }}e^{-{\left(D_{\min}^{i}/n\sigma \right)}^{2}}\right)}^{-1}, \end{array}$$

where *σ* is the measurement standard deviation of sensor in [*t*_*p*_, *t*_check_] and *n* is the number of the hypothetical source position.

A distribution map of location probability can be obtained after the set of location probabilities, (*P*_*r*_^1^, *P*_*r*_^2^,…, *P*_*r*_^*i*^,…, *P*_*r*_^*n*^), has been calculated by using (8). This map is directly able to show where the source appears with max location probability. We denoted max⁡(*P*_*r*_^*i*^) as the global optimal objective function and let *P*_*r*_^*q*^ = max⁡(*P*_*r*_^*i*^), where the superscript *q* is the node corresponding to the max⁡(*P*_*r*_^*i*^).

After *P*_*r*_^*q*^ has been searched, the source can be located at *p*_*q*_; that is, (8) is used for the source location. Hence, the global optimal source parameter is (*p*_*q*_, *t*_*e*,opt_^*q*^, *S*_opt_^*q*^) and this is the final identification result for all hypothetical positions, (*p*_1_, *p*_2_,…, *p*_*i*_,…, *p*_*n*_).

## 4. Identifiability

The presented identification method is relatively effective because only (1) is used for estimating a source reversely. But its performance of identification could be acceptable only when the assumptions about $\overset{⃑}{u}$ and *ρ* are satisfied very well. If not, then an identification error will be generated. In this section, the impact of $\overset{⃑}{u}$ and *ρ* on the performance of identification will be discussed in detail in order to obtain the application conditions.

### 4.1. Velocity Field

The velocity field, $\overset{⃑}{u}$, has an important impact on the identification of pollutant source. In our analysis, $\overset{⃑}{u}$ is assumed to be large enough and remains unchanged or little changed in the whole process, so the source emission or other factors should not change velocity field greatly.

In order to test the change of velocity fields before and after a source happening, we define a synergy degree of velocity fields to quantitatively discuss how to satisfy the above velocity field assumption according to the field synergy principle [30].

For two scalar fields of the same variable *φ* in the same space, denoted by the field *A* and *B*, we define a nonnegative real number Φ as the synergy degree of the field *A* and the field *B* for the same variable. Φ is expressed as a percentage:

(9) $$\begin{array}{c} \Phi =\max\left\{\frac{\varphi_{B}\left(x,y,z\right)-\varphi_{A}\left(x,y,z\right)}{\max\left\{\left|\varphi_{A}\right|\right\}}\right\}, \end{array}$$

where *φ*_*B*_(*x*, *y*, *z*) is the value of variable *φ*at (*x*, *y*, *z*) position in the field *B*; *φ*_*A*_(*x*, *y*, *z*) is the value of *φ*at (*x*, *y*, *z*) in the field *A*; and max⁡{|*φ*_*A*_|} expresses the maximum absolute value of the scalar *φ* in the field *A*.

If *φ* is a vector, take its components in every axis direction and obtain their corresponding synergy degrees. The largest component of synergy degrees will be the synergy degree of vector *φ* between the field *A* and the field *B*.

${\overset{⃑}{u}}_{A}$ and ${{\overset{⃑}{u}}_{B}}_{}$ are the velocity fields before and after a sudden source appears. We can obtain the synergy degrees of velocity fields with the definition of (9). If Φ ≈ 0, then ${\overset{⃑}{u}}_{A}\approx {{\overset{⃑}{u}}_{B}}_{}$, which represents the velocity field, is not changed greatly; If Φ > 0, then ${\overset{⃑}{u}}_{A}\neq {{\overset{⃑}{u}}_{B}}_{}$, which represents the velocity field, is changed. The greater the value of Φ is, the greater the velocity field will be changed.

**Figure 2:**
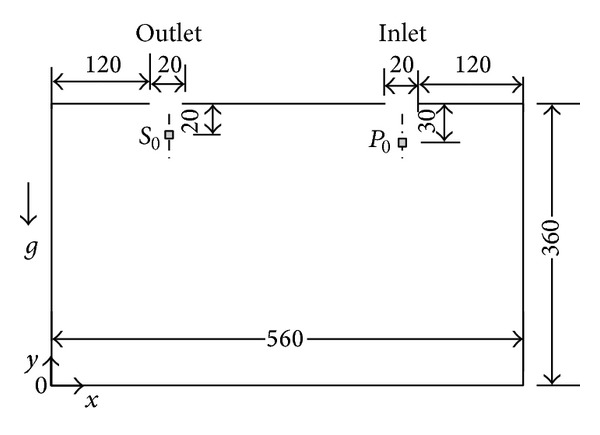
Size of the simulation space (unit: mm).

One of the prerequisites to using our identification method is ${\overset{⃑}{u}}_{A}\approx {{\overset{⃑}{u}}_{B}}_{}$; hence, Φ should be required to be as small as possible. In order to discuss the impact of *v*_in_ or $\overset{⃑}{u}$ on Φ, we use a 2D simulation case, as shown in Figure 2, for the analysis.

It is a rectangular enclosed space, 560 mm long and 360 mm wide, with a 20 mm wide inlet and a 20 mm wide outlet. There is a pollutant source at *P*_0_, and it is a 10 mm × 10 mm square pollution source. There is a pollutant sampling point at *S*_0_. The direction of gravity is the direction of −*y*.

In our study, CO_2_ is the aim pollutant. Its molecular weight is larger than air. The source begins to release the CO_2_ pollutant with constant emission strength at 0 second after the velocity field has become steady. The source emits a trace amount of the pollutant continuously.

Four cases with different *v*_in_ and *S* were analyzed. According to the above definition, we can obtain the synergy degrees of velocity fields before (*t* = 0) and after a sudden source appears in the gravity field space for the case in Figure 2 with different inlet velocity and source conditions. They are listed in Table 1.

From Table 1, we can see that

1. the synergy degree of velocity fields Φ will increase with the decrease of *v*_in_. For example, when *S* = 0.5 kg/(m^2^·s), Φ is changed from 1.47% to 13.12% corresponding to *v*_in_ changing from 1 m/s to 0.3 m/s.
2. If we assume that the application condition for the source identification is Φ < 11% (in fact, Φ should be as small one as possible). Φ = 10.43% in Case 2, in which *v*_in_ = 0.5 m/s and *S* = 0.5 kg/(m^2^·s), and it satisfies the assumption condition of Φ < 11%. While for the same *S* and a smaller inlet velocity (*v*_in_ = 0.3 m/s) in Case 3, its Φ = 13.12% and it does not satisfy Φ < 11%. But if *S* becomes smaller, for example, *S* = 0.25 kg/(m^2^·s) in Case 4 with *v*_in_ = 0.3 m/s, then its Φ = 9.34% and it satisfies Φ < 11% again. So, there is a close relationship between *v*_in_ and *S*. Therefore, a larger inlet velocity is more ideal to satisfy the assumption condition of application than a smaller one and will ensure that the source could be identified.

### 4.2. Momentum Equation

In this section, we will discuss in what situation the impact of the momentum equation can be ignored.

If only considering the *z*-axis direction (*u*_*x*_ = *u*_*y*_ = 0), the one-dimensional flow velocity *u*_*z*_ is taken as a fixed value and *μ* is very small for the air and then (2) can be written as follows:

(10) $$\begin{array}{c} \frac{\partial \rho }{\partial t}=-\frac{1}{u_{z}}\frac{\partial \left(\rho u_{z}\right)}{\partial z}-\frac{1}{u_{z}}\left(\frac{\partial p}{\partial z}+\rho g\right). \end{array}$$

According to the assumptions about $\overset{⃑}{u}$ and *ρ*, we know that the absolute value of −1/*u*_*z*_ is smaller when *u*_*z*_ is greater; thus, the influence of the second term on the right (gravity and air buoyancy) becomes weaker. If we assume that *ρ* remains approximately uniform in the space, then the first term on the right of (10) can be ignored. Hence, the flow velocity and the amount of emitted pollutant are the key factors for effectively weakening the influence of the momentum equation.

We denote *C* ^*t*^ and *C* ^*i*^as the true concentration and the calculated concentration at *S*_0_ in Figure 2, respectively. *C* ^*t*^ can be obtained by solving the simultaneous equations of continuity, advection-dispersion, momentum, and energy equations. *C* ^*i*^ can be calculated by only solving (4). Because it is easier and more efficient to calculate *C* ^*i*^ than to calculate *C* ^*t*^, we will compare *C* ^*i*^ with *C* ^*t*^ and determine in what situation is it more suitable to use *C* ^*i*^ instead of *C* ^*t*^ for the identification.

**Figure 3:**
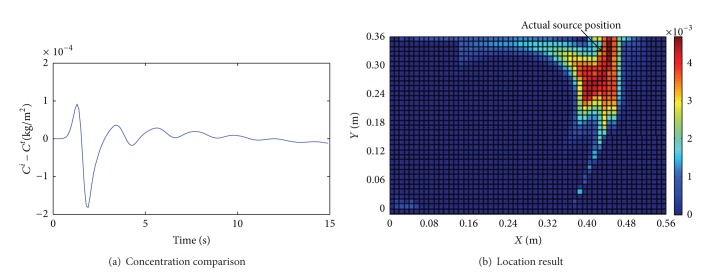
Results for Case 1 (*v*_in_ = 1 m/s, *S* = 0.5 kg/m^2^·s, Φ = 1.47%).

The above four cases in Table 1 are also used again for the comparison. (*C* ^*i*^ − *C* ^*t*^) is used to show the difference of *C* ^*i*^ and *C* ^*t*^. Figures 3(a)~6(a) show the comparison results of (*C* ^*i*^ − *C* ^*t*^) for the four cases. We let *C* ^*t*^ stand for the virtual measured concentration at *S*_0_ with an observed noise. We assume that the source is unknown and estimate it by using the presented method. Figures 3(b)~6(b) show the location results for the four cases. The deep red region indicates higher location probability and the blue region indicates that zero location probability.

We draw some conclusions from Figures 3~6.

**Figure 4:**
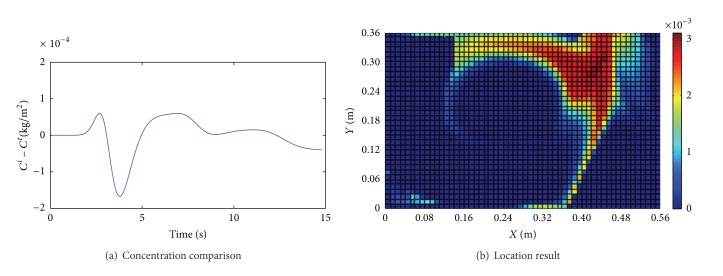
Results for Case 2 (*v*_in_ = 0.5 m/s, *S* = 0.5 kg/m^2^·s, and Φ = 10.43%).

**Figure 5:**
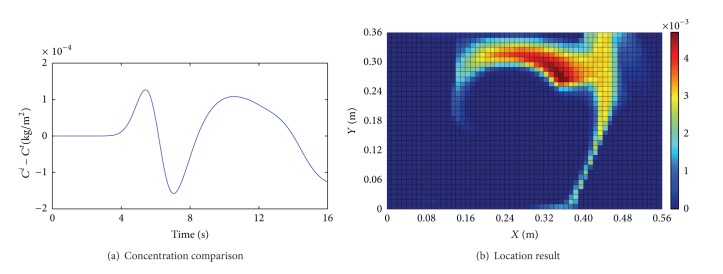
Results for Case 3 (*v*_in_ = 0.3 m/s, *S* = 0.5 kg/m^2^·s, and Φ = 13.12%).

**Figure 6:**
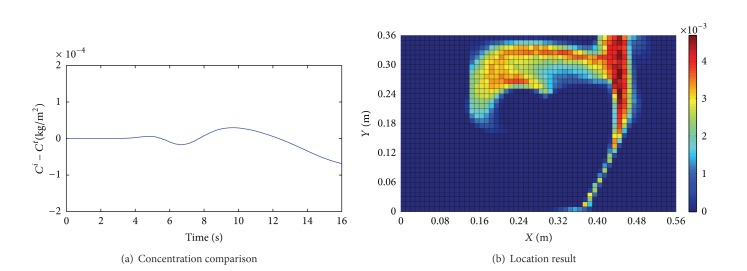
Results for Case 4 (*v*_in_ = 0.3 m/s, *S* = 0.25 kg/m^2^·s, and Φ = 9.34%).

1. The difference of *C* ^*t*^ and *C* ^*i*^ is very small in most time when the inlet velocity is *v*_in_ ≥ 0.5 m/s, as shown in Figures 3(a)~4(a). This implies that the influence of the momentum equation can be negligible. In this situation, we can use *C* ^*i*^ instead of *C* ^*t*^ for identification. The location results in Cases 1 and 2 can be acceptable because the deep red region is concentrated near the inlet and near the actual source position, as shown in Figures 3(b) and 4(b). But the difference of (*C* ^*i*^ − *C* ^*t*^) and Φ are smaller in Case 1 than Case 2; then the location result in Case 1 is better than the one in Case 2.
2. When *v*_in_ < 0.5 m/s, the difference between *C* ^*t*^ and *C* ^*i*^ becomes large as shown in Figure 5(a). This implies that the momentum equation has played a leading role in the CO_2_ transmission and can not be ignored. We can not obtain a reasonable location result, as shown in Figure 5(b). However, if the source strength becomes tracer, for example, *S* = 0.25 kg/(m^2^·s) in Case 4, then we can obtain a more reasonable location result, as shown in Figure 6(b). This is because Φ in Case 4 is equal to 9.34% and it is less than the one in Case 3.

### 4.3. Application Conditions

According to the above analyses, we know the following.

1. The value of the inlet velocity or the velocity field is the key to satisfy the application assumptions of the presented method. An inlet velocity should be large enough to ensure that the source is effectively identified.
2. The inlet velocity and the source emission strength should be matching. The source emission process should not disturb the velocity field. So, a source with strong emission strength could be located in a large velocity field, but it cannot be located accurately in a small velocity field. For a small velocity field, a source needs to be one with small emission strength to be estimated accurately.

Therefore, the identification method is actually suitable for estimating a trace continuous pollutant source in a steady velocity field. In the actual application, we can refer to this idea to analyze a studied situation and determine its application conditions.

## 5. Experiment Study

### 5.1. Experimental System

An experimental device was set up to test the proposed method. CO_2_ is used as the aim pollutant. CO_2_ gas injected into the enclosed space from a point is supplied by a compressed gas cylinder through a pressure relief valve and a mass flow controller. A QIC-20 mass spectrometer made by the UK company Hiden Analytical Ltd. was used to measure CO_2_ concentrations in real time at the sampling point.

**Figure 7:**
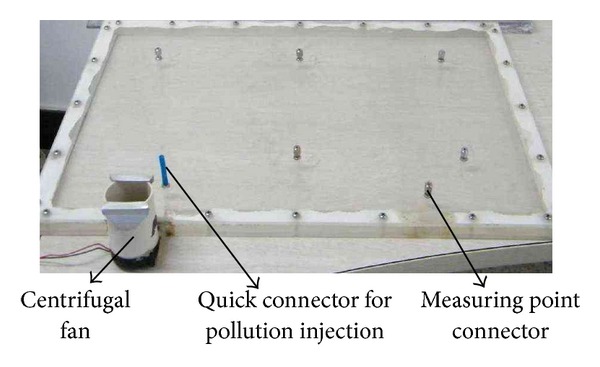
Experimental device photo of 2D space.

The photo of the enclosed space is shown in Figure 7 and its size is given in Figure 2. It is a flat cavity topped and bottomed by two plexiglass flat panels and four-side sealing strips. Due to the thickness of the enclosed space being only 8 mm and relatively very thin, the flow velocity, which is formed by a small centrifugal fan, in the thickness direction can be considered approximately zero; thus, the flow field is close to a 2D case.

### 5.2. Source Identification

**Figure 8:**
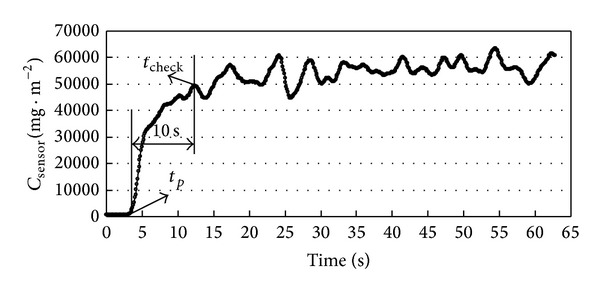
Measured CO_2_ concentration data.

During the experiment, the inlet velocity was 1.68 m/s. CO_2_ concentration curve measured at *S*_0_ is shown in Figure 8 when the CO_2_ was suddenly injected from *P*_0_. The source emission strength was constant and its actual emission strength was 16.37 mg/s.

For the experimental space in Figure 7, we know that

1. *v*_in_ = 1.68 m/s and it is larger than 1.0 m/s of Case 1 in Table 1. At the same time, its Φ is larger than 1.47% and $\overset{⃑}{u}$ is steady. Hence the assumption of $\overset{⃑}{u}$ is satisfied very well;
2. *S* = 16.37 mg/s and it is less than 50 mg/s in Case 1. This means that the emission amount of source is small and the assumption of *ρ* is satisfied.

Therefore, this experimental situation satisfies the application conditions in Section 4.3.

We apply the identification method to identify the source reversely by using the experimental data in Figure 8. The other parameters are *t*_*p*_ = 3.3 s, *t*_check_ − *t*_*p*_ = 10 s, *C*_0_ = 655 mg/m^3^, and Δ*t* = 0.1 s.

**Figure 9:**
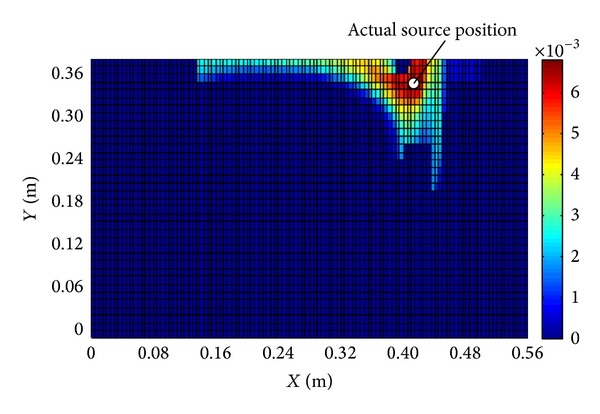
Distribution map of location probability for the experimental case.

The estimated emission strength is 20.862 mg/s, and the final distribution map of location probability is shown in Figure 9. From Figure 9, we know that the position having the largest location probability is very near to the actual pollutant source position.

## 6. Conclusions

A method to identify a trace continuous source in a steady flow field was proposed in this paper. The identification method is realized by globally searching the optimal values with the objective function of maximum location probability.

In order to ensure the performance of identification, the assumptions about $\overset{⃑}{u}$ and *ρ* are discussed in detail and the application conditions for the identification method are obtained. A synergy degree of velocity fields Φ is introduced to analyze the application conditions of velocity field quantitatively. The value of Φ should be as small as possible in order to satisfy this assumption of the velocity field. Based on this premise, we draw the following application conditions from the simulation studies:

1. the value of the velocity field (or the inlet velocity) is one key factor. A larger $\overset{⃑}{u}$ is more ideal in satisfying the application conditions;
2. The source emission strength *S* is another key factor in addition to $\overset{⃑}{u}$. The source emission process can not disturb $\overset{⃑}{u}$ greatly and *S* and $\overset{⃑}{u}$ should be matching each other. Therefore, the identification method is suitable for estimating a trace continuous pollutant source in a steady velocity field.

In order to investigate the practical application effect of the above studies, the experimental system was set up and subsequently a sudden pollution experiment was conducted. The presented identification method was used. The studies show that the sudden trace continuous source can be successfully located when the experimental situation satisfies the application conditions.

## Figures and Tables

**Table 1:** Values of Φ in different simulation cases.

Case	*v*_in_ (m/s)	*S* kg/(m^2^·s)	Φ (%)
Case 1	1.0	0.5	1.47
Case 2	0.5	0.5	10.43
Case 3	0.3	0.5	13.12
Case 4	0.3	0.25	9.34
